# Intermittent parathyroid hormone enhances the healing of medication-related osteonecrosis of the jaw lesions in rice rats

**DOI:** 10.3389/fmed.2023.1179350

**Published:** 2023-06-19

**Authors:** E. J. Castillo, J. M. Jiron, C. S. Croft, D. G. Freehill, C. M. Castillo, J. Kura, J. F. Yarrow, I. Bhattacharyya, D. B. Kimmel, J. Ignacio Aguirre

**Affiliations:** ^1^Department of Physiological Sciences, University of Florida, Gainesville, FL, United States; ^2^VA Medical Center, Research Service, Gainesville, FL, United States; ^3^Department of Oral and Maxillofacial Diagnostic Sciences, College of Dentistry, University of Florida, Gainesville, FL, United States

**Keywords:** anti-resorptives, MRONJ, periodontitis, PTH, healing

## Abstract

Medication-related osteonecrosis of the jaw (MRONJ) is a potentially severe adverse event in patients treated with antiresorptives. Management of MRONJ is challenging, and no non-antibiotic, established medical treatment exists. Intermittent parathyroid hormone (iPTH) has been used off-label to treat MRONJ with favorable results. However, its medical efficacy has rarely been substantiated in clinical or preclinical experiments. Using a validated rice rat, infection-based model of MRONJ, we evaluated the effects of iPTH on established MRONJ. We hypothesize that iPTH contributes to MRONJ resolution by enhancing alveolar bone turnover and healing oral soft tissues. Eighty-four rice rats began a standard rodent chow diet at age 4 weeks to induce localized periodontitis. Rats were simultaneously randomized to receive saline (vehicle, VEH) or zoledronic acid (ZOL, 80 μg/kg IV) every 4 weeks. Oral exams were conducted bi-weekly to assign a gross quadrant grade (GQG, 0–4) to evaluate any lesion at the lingual aspect of the interdental space between maxillary molar (M2) and M3. 14 of 20 VEH-treated rice rats (70%) developed maxillary localized periodontitis with GQG 2–3 after 30 ± 10 weeks of saline. Additionally, 40 of 64 ZOL-treated rice rats with periodontitis developed MRONJ-like lesions after 30 ± 10 weeks of ZOL treatment. Rice rats with localized periodontitis or MRONJ-like lesions were treated with saline or iPTH (40 μg/kg) subcutaneously (SC) 3 times/week For 6 weeks until euthanasia. We found that iPTH -treated ZOL rats had a lower prevalence of MRONJ (*p* < 0.001), with lower severity extent of oral lesions (*p* = 0.003) and percentage of empty osteocyte lacunae (*p* < 0.001). ZOL rats treated with iPTH displayed a higher osteoblast surface (*p* < 0.001), more osteoblasts (*p* < 0.001), higher osteoclast surface (*p* < 0.001) and more osteoclasts (*p* = 0.002) at alveolar bone surfaces than ZOL/VEH rats. Greater gingival epithelial thickness and epithelial cell proliferation rate was found in the oral mucosa and gingiva of ZOL/PTH rats than in ZOL/VEH rats (*p* < 0.001). Our data suggest that iPTH is an efficacious non-operative medicinal therapy that accelerates oral healing and enhances the resolution of MRONJ lesions in ZOL-treated rice rats.

## Highlights

– iPTH reduces the burden of disease in ZOL-treated rice rats with established MRONJ lesions
– iPTH accelerates bone remodeling of the necrotic alveolar bone of MRONJ lesions
– iPTH accelerates soft tissue healing of MRONJ lesions.

## Introduction

Medication-related osteonecrosis of the jaw (MRONJ) is a potentially severe adverse event with the following features: (1) oral lesions with exposed bone or bone that can be probed through an intraoral or extraoral fistula(e) in the maxillofacial region that has persisted for more than 8 weeks; (2) affected patients that have current or previous treatments with powerful antiresorptives (pARs) alone or in combination with immune modulators or antiangiogenic medications; and (3) no history of radiation therapy or metastatic disease to the jaws (1, 2).

MRONJ is common in patients with cancer (1.8–5% incidence) and rare in patients with osteoporosis (0.01–0.03% incidence) (1–4). pARs, including nitrogen-containing bisphosphonates [N-BPs, e.g., zoledronic acid (ZOL)] and anti-RANKL antibodies (e.g., denosumab), are prescribed to manage hypercalcemia and bone metastases in patients with cancer and to prevent fragility fractures in patients with osteoporosis (1, 2, 5–10).

Both clinical and preclinical studies demonstrate that for most MRONJ to occur, the concurrent administration of pARs (systemic risk factor) is required along with the co-existence of a local oral risk factors such as inflammatory dental disease (e.g., periodontitis, periapical infection), tooth extraction, or ill-fitting removable partial prostheses (1, 2, 7, 11–22). Although implementing prophylactic treatment modalities, including dental screenings and regimented oral health surveillance for at-risk patients, has reduced disease incidence, MRONJ remains a significant problem. Furthermore, once patients develop MRONJ, clinical management can be challenging, and the outcome can be difficult to predict and occasionally problematic (7, 23–27).

The American Association of Oral and Maxillofacial Surgeons (AAOMS) developed a staging system based on the severity of clinical symptoms and findings. It streamlines the evaluation process and identifies management/treatment strategies for each stage of MRONJ based on expert opinion (1). The management/treatment strategies include both non-operative therapies (e.g., antimicrobial rinses, systemic antibiotics and improved oral hygiene) and operative therapies (e.g., sequestrectomy, resection) (1, 28–34). Operative therapies have been reported as a viable option with high success rates for all stages of the disease (35–37). However, non-operative therapies remain a treatment option for MRONJ, particularly in stage 1 MRONJ, especially where significant comorbidities preclude operative treatment (1, 38, 39). There is little evidence to suggest that platelet-rich plasma, low-level laser irradiation and hyperbaric oxygen therapy lead to MRONJ resolution (1). However, PTH (teriparatide, Forteo [Eli Lilly Co.]), a recombinant peptide consisting of the 1–34 amino acids of human PTH, has emerged as a promising option due to its potential to accelerate the healing of affected jaw bones by enhancing bone turnover and speeding the removal of necrotic bone (40, 41). Indeed, teriparatide has been successfully used off-label as an adjunct medication for MRONJ treatment in patients with osteoporosis (31–34, 42). Teriparatide was clinically associated with increased bone regeneration, lesion size reduction, and a higher resolution rate of MRONJ (32, 34, 42, 43). Most preclinical studies substantiated the positive effect of intermittent (i) administration of PTH as a preventive treatment for MRONJ (44–49).

In contrast, only a few investigated the effect of iPTH on established MRONJ, evaluating only radiographic and/or histopathologic features (50, 51). Despite the contributions of the above studies, knowledge gaps still exist on the role that iPTH could play in the reversal of necrotic alveolar bone, low alveolar bone remodeling, and the healing of MRONJ lesions. No comprehensive bone histomorphometric analysis has yet been conducted to assess the effects of iPTH on alveolar bone formation and resorption in and around MRONJ lesions. In addition, after MRONJ is established, a secondary disturbance of the associated oral soft tissue homeostasis occurs. Indeed, disruption of the oral mucosal integrity accompanies the bone exposure, infection and inflammation observed in MRONJ lesions (52, 53). In line with this notion, N-BPs were reported to suppress epithelial cell proliferation, increase apoptosis, induce mucosal thinning and inhibit oral mucosal wound healing (54–57). On the contrary, PTH and PTH-related peptides (PTHrp) reduce inflammation and promote wound healing in the femur, tibia and periodontium (58–61). Furthermore, PTH and PTHrp affect the proliferation of epithelial and mesenchymal cells but with opposite effects depending on cell and tissue types (61–69). The effect of iPTH on the oral epithelium is undefined. Furthermore, few studies have shown that iPTH decreases the number of inflammatory cells, including neutrophils and CD3^+^ T cells in periodontitis models (70–73). However, the effects of iPTH on the inflammatory response in oral soft tissues associated with MRONJ lesions have rarely been investigated (50). Thus, in this study using the well-established rice rat MRONJ model (21, 74, 75), we investigated the effects of iPTH on both the alveolar bone of MRONJ lesions, using static and dynamic histomorphometry; and the associated oral soft tissues, using morphometry and immunohistochemistry. We hypothesized that iPTH assists healing of MRONJ lesions by accelerating the resorption of necrotic alveolar bone, enhancing alveolar bone formation, promoting gingival epithelium regeneration and reducing inflammation in both the hard and soft tissues of an MRONJ lesion.

## Materials and methods

### Animal care and management

Rice rats were generated in-house using a monogamous continuous breeding system (76). Care and management were undertaken as before (20, 21). The Animal Care Services at the University of Florida (UF) is an AAALAC-accredited animal care and use program. The experimental protocol (#202008453) was approved by the UF Institutional Animal Care and Use Committee (IACUC).

### Study design

60–80% of the rice rats fed a standard (STD) rodent chow [Envigo Teklad LM-485 (irradiated 7,912) Rodent Diet; Tampa, FL, United States] from age 4 weeks develop a localized form of periodontitis by ages 16–22 weeks, with severity gross quadrant grades (GQG) that range from 1 to 3 on a grading scale of 0–4 (Supplemental Table S1) (77). When rice rats developing localized periodontitis are simultaneously treated with an oncology dose of ZOL, MRONJ lesions develop at the site of localized periodontitis in 70–100% of rats after 18–30 weeks treatment (21, 74, 78). In contrast, MRONJ does not develop in rice rats with localized periodontitis in the absence of ZOL. In the current study, we used the ZOL rice rat model of MRONJ to investigate the effect of iPTH on established MRONJ lesions that arose in the context of inflammatory dental disease.

Eighty-four clinically healthy [body weight (BW) ≥ 30 g and body condition score (BCS ≥3.0) (79)] rice rats of both genders (42 males/42 females), age 4 weeks, were fed the STD diet to induce localized periodontitis. Special efforts were made to distribute littermates and genders equally among the experimental groups and subsets. Animals were randomized at age 4 weeks to receive either saline (*n* = 20; 10 males/10 females) or an oncologic equivalent dose of ZOL (80 μg/kg BW; *n* = 64; 32 males/32 females) intravenously (IV) every 4 weeks until the end of the study as previously (18, 20, 21, 80). ZOL was provided by Novartis Pharma AG (Basel, Switzerland), dissolved in sterile saline (pH 7.2, 0.2 mg/ml) and injected into the tail vein at 0.4 ml/100 g BW.

Supplemental Figure S1 depicts the study design and experimental groups. Sixteen of the 20 VEH rice rats (80%) developed maxillary localized periodontitis with GQGs 2–3 at age 34 ± 10 weeks. The VEH rice rats with localized periodontitis were further randomized by GQG into two subsets: one (males *n* = 4 and females *n* = 4) received 0.9% saline subcutaneously (SC) 3 times/week for 6 weeks (*VEH/VEH*), and the other (males *n* = 4 and females *n* = 4) received human PTH [1–34; teriparatide; 40 μg/kg (Bachem, Torrance, CA, United States)] SC 3 times/week. for 6 weeks (*VEH/PTH*). Forty of 64 (~63%) rice rats with localized periodontitis simultaneously treated with ZOL developed oral lesions compatible with MRONJ (MRONJ-like lesions) after 30 ± 10 weeks of ZOL treatment (21, 74, 78). The 40 ZOL-treated rats with MRONJ-like lesions were further randomized by GQG into two subsets of 20 rats. One subset (males *n* = 11; females *n* = 9) received saline subcutaneously (SC) 3 times/week for 6 weeks (*ZOL/VEH*). The other (males *n* = 11; females *n* = 9) received PTH SC 3 times/week for 6 weeks (*ZOL/PTH*). All groups had almost equally distributed genders and GQG severity. Bi-weekly oral exams (66), BW measurements and ZOL or saline administration continued through the end of the study. Rice rats with BW loss or BCS deterioration were monitored daily and offered diet gel and supplemental fluids. Gender effects were not investigated since MRONJ prevalence is not affected by gender. Rats were injected subcutaneously with the fluorochromes demeclocycline (15 μg/kg) and calcein (15 μg/kg) 7 and 2 days before euthanasia, respectively, to enable dynamic bone histomorphometric data collection. After 6 weeks of iPTH or saline, rats were euthanized, and jaws were collected for gross, histologic, static and dynamic histomorphometric analyses.

### *In vivo* oral exams and definition of MRONJ-like lesion

Rice rat oral exams began at age 4 weeks. They were performed bi-weekly and continued through the end of the study. Briefly, isoflurane-anesthetized rice rats were presented randomly to calibrated investigators (EC, JA) who were blind to the treatment group and any previous measurements on the animal. Oral exams were performed with 4X dental loupes to assess the state of each oral maxillary lesion, particularly at the lingual aspect of the maxillary interdental M2M3 region, where localized periodontitis develops (74, 78). In addition, each maxillary quadrant was assigned a GQG indicative of lesion severity (Supplemental Table S1) (21, 78). Because oral lesions must be confirmed histopathologically to formally diagnose MRONJ, we use the term “MRONJ-like” for oral lesions that displayed similar gross oral features as those seen in rice rats with histopathologically-confirmed MRONJ diagnoses in previous studies (21, 74, 78). These characteristic gross features include a continuous recession/ulceration of the gingiva at the lingual aspect of the maxillary M2-M3 region, with gingival swelling and alveolar or palatal bone exposure. In addition, lesions may extend mesially to the lingual aspect of the middle of M1 and distally to the distal of M3.

### Euthanasia and tissue collection

Rice rats were euthanized by CO_2_ inhalation, followed by cervical dislocation. Blood was collected by cardiac puncture prior to cervical dislocation. The serum was separated and stored at -20^o^ C. Jawbones were excised and trimmed, and high-resolution photographs of the four quadrants were taken as previously (21). The four quadrants were fixed at 4°C in 4% paraformaldehyde for 48 h and then transferred to 70% ethanol. The hemi-maxillae with oral lesions were embedded undecalcified in modified methyl methacrylate to conduct bone histomorphometry as previously (18, 81). Contralateral hemi-maxillae and left mandibles were decalcified, and paraffin-embedded sections were obtained for different histologic and immunohistochemistry evaluations (20, 74, 78). Left femurs were assessed independently by peripheral quantitative computed tomography (pQCT) to verify the efficacy of ZOL treatment.

### Gross *ex vivo* analysis of maxillary lesions

The *ex vivo* oral exam was performed to examine and score the severity of the jaw lesions using high-resolution photographs of maxillae and mandibles as previously (21, 74, 78). All photographs were presented for GQG scoring in a randomized order to treatment-blinded investigators (EC; JA) who had no knowledge of previous oral exam results. MRONJ-like lesions were defined using the same *in vivo* oral exam criteria. Gross maxillary oral lesion area (Le.Ar, mm^2^) and total maxillary area (Tt.Mx.Ar, mm^2^) were measured in quadrants with GQG ≥ 2 with the outline tool of the AxioVision SE64 Rel software version 4.9.1 (Carl Zeiss, Germany) as previously (21). Le.Ar was expressed as a percentage of Tt.MxAr (=100 * Le.Ar/Tt.Mx.Ar).

### MicroCT analysis of maxillae

MicroCT scanning was performed on the excised maxillae of *ZOL/VEH* (*n* = 6) and *ZOL/PTH* (*n* = 6) rice rats with MRONJ-like lesions (GQG 2–4) to assess the effects of iPTH on radiographic variables in the alveolar bone associated with MRONJ lesions. Images were acquired using the following parameters: 80 kVP/120 μA, 0.5 mm aluminum filter, 2 k camera resolution, 9.58 μm voxel size, 0.5° rotation step, and 360° tomographic rotation. Cross-sectional images were reconstructed using a filtered back-projection algorithm (Bruker Skyscan, NRecon, Kontich, Belgium) as previously (82) and per recommendations of the American Society of bone and Mineral Research (83). The 2D images were aligned identically in the mesiodistal and axial planes, with all molars simultaneously visible. A rectangular ROI of 1.50 mm × 3.0 mm apical to the furcation of M2 was applied to all samples, and analysis was done on 65 consecutive slices excluding all molars.

### Preparation of sections from undecalcified maxillae

Maxillae were sectioned, and the crowns of the molars were ground down with a dremel to remove the enamel. Samples were then dehydrated, infiltrated, and embedded undecalcified in methyl methacrylate (MMA; Sigma Chemical Co, St. Louis, MO, United States) as previously (18). Four- and eight μm thick sections were obtained with a Leica/Jung 2,265 (Leica Biosystems Inc. Buffalo Grove, IL, United States) microtome. The four-μm sections were stained with von Kossa and counterstained with tetrachrome (Polysciences Inc., Warrington, PA) for structural and static histomorphometry and with toluidine blue to assess the number of empty osteocyte lacunae, confirmation of MRONJ lesions and its prevalence (18). The eight-μm sections were left unstained for dynamic histomorphometric measurements (see below).

### Assessment of MRONJ and quantification of necrotic bone

Exposed bone was verified in undecalcified sections by the absence of overlying gingival epithelium and lamina propria (18, 20, 21). Necrotic bone was identified using: (1) a pattern recognition approach (84–86) and (2) specific criteria from preclinical MRONJ studies that require bone matrix containing ≥10 adjacent lacunae that were either empty or contained pyknotic osteocyte nuclei or cellular debris (87, 88). Two observers (EC and JA) surveyed all levels independently. When their diagnoses differed, an agreement was reached by reviewing relevant slides concurrently. Data collection concerning osteocyte lacunae was performed at 200× magnification in sections within a 0.15–0.25 mm^2^ region of interest containing localized periodontitis or MRONJ lesions. The total number of osteocyte lacunae and number of empty lacunae were counted to calculate the percentage of empty osteocyte lacunae and the number of empty lacunae per bone area (#/mm^2^) as previously (20, 21, 78).

### Bone histomorphometry of maxillary alveolar bone

**Figure 1:**
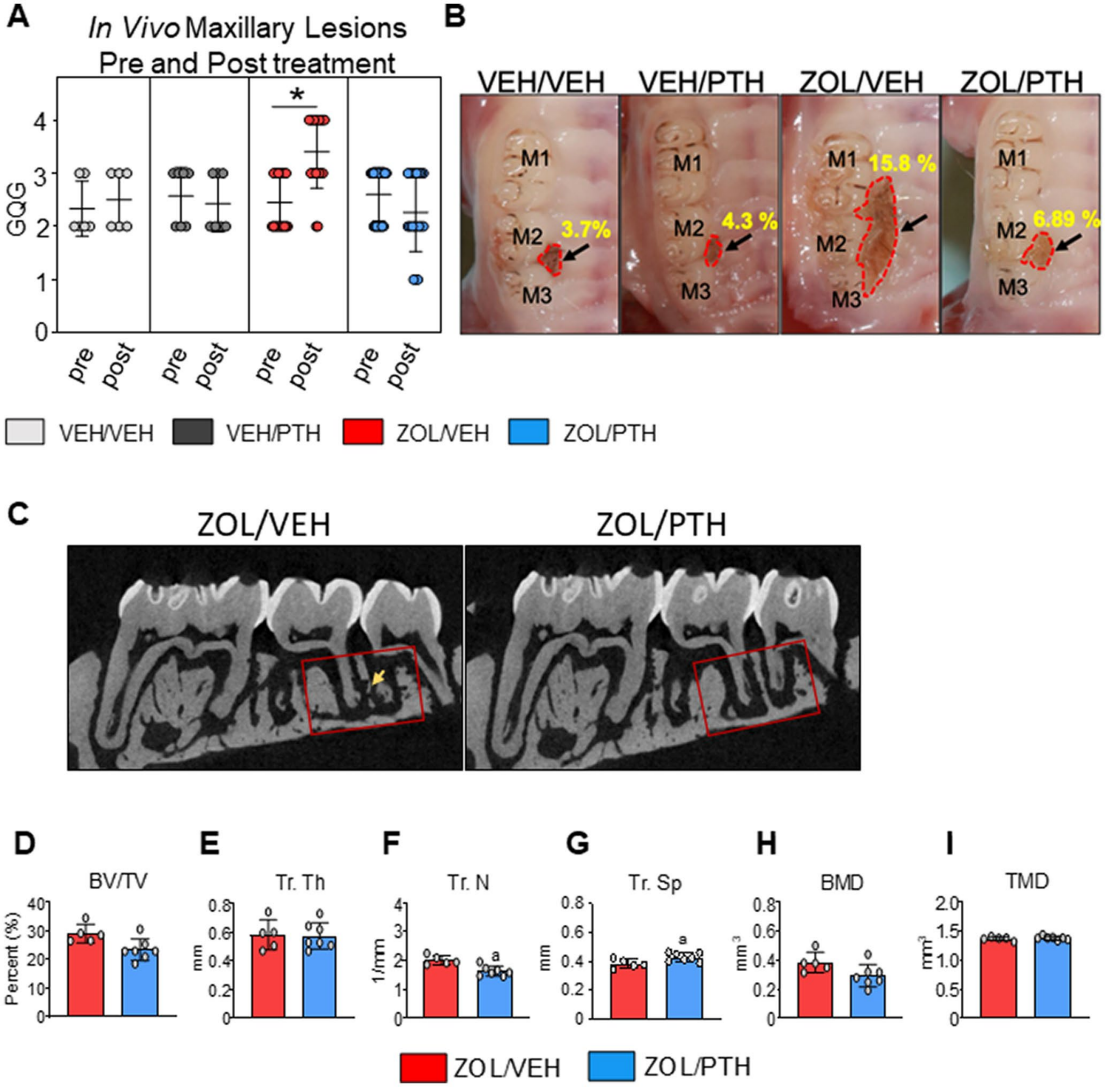
Gross oral lesions and MicroCT features were found in rats with MRONJ-like lesions treated with either VEH or iPTH. **(A)** The severity of oral lesions (GQG) was assessed by *in vivo* oral exams at the start and end of treatment (6 weeks). The scatter dot plot graph includes mean ± SD. *Signifies significant differences between pre- and post-treatment readings (*p* < 0.01). **(B)** Representative high-resolution photomicrographs of rice rats of each group depicting maxillary lesions at the M2M3 interdental region at necropsy. The black arrow points to the lesion. The red dotted line demarcates the lesion area. VEH/VEH and VEH/PTH rice rats have small and confined oral lesions. Lesions were significantly larger in ZOL/VEH rats than in all other groups, with ulceration and recession of the gingiva extending along the three molars and toward the palatal midline. **(C)** 2D Micro CT slices of maxillae in the mesiodistal plane showing the region of interest location (red box) in the M2M3 interdental region where MRONJ-like lesions became established, and rats were treated with either saline or ZOL for 6 weeks. The ROI extends from the distal aspect of the M1 distal root to the mesial of the M3 distal root. A rectangular ROI of 1.50 mm × 3.0 mm apical to the furcation of M2 was applied to all samples. Sequestered bone can be seen in the ZOL/VEH rat (yellow arrow) but not in the ZOL/PTH rat. **(D)** Bone volume (BV/TV). **(E)** Trabecular thickness (Tr. Th), **(F)** Trabecular number (Tr. N) and **(G)** Trabecular separation (Tr. Sp). **(H)** Bone mineral density (BMD). **(I)** True mineral density (TMD). Error bars indicate Mean ± SD. In figures **D-I**, superscript a indicates significant differences from ZOL/VEH rats (*p* < 0.05).

Static and dynamic bone histomorphometry was performed at two regions of interest (ROI) to assess the effects of iPTH on alveolar bone remodeling. ROI 1 is localized at the maxillary interdental region between molar (M) 2 and M3, the specific site of the oral lesions (Figure 1C). ROI 2 is localized far distal to M3, an unaffected adjacent site not involved in the oral lesion. Evaluating histomorphometric variables at these two sites provided insight into the effects of iPTH at both affected and unaffected oral sites. Static structural bone parameters included alveolar bone volume (BV/TV), trabecular thickness (Tb.Th), trabecular number (Tb.N), and trabecular separation (Tb.Sp). Static cellular bone parameters included osteoblast number per alveolar bone perimeter (N.Ob/B.Pm), osteoblast surface (Ob.S/BS, %), osteoclast number per alveolar bone perimeter (N.Oc/B.Pm) and osteoclast surface (Oc.S/BS, %). Dynamic histomorphometric parameters included single-labeled surfaces (sLS/BS), double-labeled surfaces (dLS/BS), mineralizing surface (MS/BS, %), mineral apposition rate (MAR, μm/day), and bone formation rate (BFR/BS, mm^3^/mm^2^/day), were assessed analyzing fluorochrome labels under ultraviolet illumination. In rice rats with maxillary tissues devoid of double-labeled surfaces, MAR was a missing value that was not considered in the calculations, and BFR/BS was reported with an imputed value of 0.1 mm^3^/mm^2^/day. Methods and measurements are based on recommendations of the Histomorphometry Nomenclature Committee of the American Society of Bone and Mineral Research (89) and our published methods (18, 81, 90, 91).

### Oral/gingival epithelium thickness and proliferation

To investigate the response of the oral/gingival to iPTH treatment, we measured epithelial thickness and quantified basal epithelial cell proliferation at two sites: (1) the M1 region of the mandible, which is a healthy region in this model, and (2) the M2M3 region of the maxilla, which represents the involved oral area (21, 77). For the mandibular M1 region, decalcified tissues were embedded in paraffin and coronally sectioned at four μm thickness. Five coronal sections 250 μm apart from each other that included lingual and buccal surfaces and the mesial or distal roots were stained with H&E and blindly analyzed using the Osteomeasure system. To analyze the maxillary M2M3 region, we used five parasagittal decalcified sections of oral lesions with GQG ≥ 2. We obtained sections 250 μm apart from each other, starting 1 mm lateral to the midline of the palate up to the buccal surface.

The gingival epithelium of the mandible was assessed at four regions: junctional, sulcular, free gingiva, and attached oral gingival epithelium. The gingival epithelial thickness of the four regions was measured with Osteomeasure software using a light microscope at ×200 magnification, averaging five different measurements per region. We investigated gingival epithelial proliferation by assessing the immunoreactivity of Ki67, a well-established cell proliferation marker (92), at the basal cell layer of each gingival epithelial region at the two sites using immunohistochemistry. Briefly, sections were deparaffinized, treated with 3% H_2_O_2_ in methanol for 10 min, and blocked for 30 min with 2% goat serum. Sections were then incubated for 2 h at room temperature with a rabbit polyclonal anti-Ki67 antibody [SP6] (ab21700; primary antibody), followed by incubations with a biotinylated goat anti-rabbit IgG (secondary antibody) and reagents of the ABC-HRP Kit (Vector Laboratories; Burlingame, CA, United States). In addition, Diaminobenzidine (Vector Laboratories; Burlingame, CA, United States) was used as chromogen and hematoxylin as a counterstaining. Gingival proliferation for each gingival region at the mandibular M1 region was expressed as a percentage of positive Ki67 basal epithelial cells of the total epithelial cells (proliferating cells/total cells ×100). The expression of Ki67^+^ epithelial cells was also evaluated at the gingiva of the involved maxillary M2M3 area. However, due to the disruption and hyperplasia of the gingival epithelium in the affected maxillary M2M3 region, the expression of Ki67^+^ epithelial cells was calculated considering the total area of the epithelium without considering the four different gingival zones. The number of proliferating cells was calculated by counting the number of Ki67+ cells/mm^2^ gingival tissue at the M2M3 interdental region (#/mm^2^).

### Quantification of T-cells and neutrophils in oral soft tissues associated with MRONJ

To give an insight into the effects of iPTH treatment on the inflammatory infiltrate associated with MRONJ lesions, T-cells and neutrophils were quantified at the maxillary ROI-1 lesion area by determining the expression of CD3 and anti-neutrophil elastase positive cells, respectively. Briefly, 4 μm-thick decalcified sections of maxillae were deparaffinized and rehydrated through xylene and graded alcohols. A rabbit polyclonal anti-CD3 antibody, present in T-cell co-receptor for CD4 and CD8 positive cells (A0452, Dako, Carpinteria, CA, 1 μg/ml) and a rabbit monoclonal anti-neutrophil elastase (EPR7479, Abcam, Boston, MA, 2 μg/ml) were used as the primary antibodies. Endogenous peroxidase was quenched using 3% hydrogen peroxide in methanol for 30 min. Heat-mediated antigen retrieval was performed by incubating slides at 95°C for 20 min in Dako Target Retrieval Solution pH 6.0 (Agilent, Santa Clara, CA, United States). Antigens were visualized with a Vectastain ABC Elite kit (Vector Laboratories, Burlingame, CA, United States). Diaminobenzidine (DAB) was used as the chromogen. Negative and positive control sections were always used. The negative controls were sections of maxillae and mandibles not incubated with the primary antibodies. The positive control sections for CD 3^+^ T cells and neutrophils were these rats’ spleen and femur diaphyseal bone marrow. Cell number and tissue area were measured using the Osteomeasure software (Osteometrics Corporation; Decatur, GA, United States). Data are expressed as the number of CD3^+^ and neutrophil elastase^+^ cells/mm^2^ of oral soft tissue area at ROI-1 (#/mm^2^).

### Statistics

Data are expressed as mean ± standard deviation. Paired T-test was used to determine the differences between the pre and post-treatment periods of the same group. The Fisher Exact test was used to determine differences in the severity of gross oral lesions and MRONJ prevalence among treatment groups. Two-way ANOVA was used to assess differences in total lesion area, empty osteocyte lacunae, bone histomorphometry parameters, gingival thickness, gingival proliferation, number of CD 3^+^ T cells and number of neutrophils. Significant differences among groups were determined by Holm-Sidak *post hoc* analysis. When linear model assumptions were not met, non-parametric Kruskal–Wallis ANOVA was used, followed by the pairwise Dunn test to assess group differences.

## Results

### General observations

All experimental animals completed the study uneventfully. No significant clinical issues associated with body weight loss, body condition score changes, or signs of pain/distress were observed (data not shown).

### Verification of systemic skeletal effects of the treatments

ZOL and iPTH-treated rats had significantly greater BMC and BMD at the femoral metaphysis and mid-diaphysis than VEH-treated control rats (Supplemental Figure S2 and Supplemental Data for Figure S2).

### Gross analysis of maxillary lesions

We found no differences in the severity grades (GQGs) of maxillary lesions between pre-and post-treatment times in VEH/VEH and VEH/PTH rats (Figure 1A). However, GQG in ZOL/VEH rats was significantly higher post-treatment than pre-treatment (*p* < 0.001). In contrast, GQG in ZOL/PTH rats was the same post-treatment as pre-treatment (*p* = 0.204) (Figure 1A).

Figure 1B depicts representative *ex-vivo* high-resolution photographs of maxillary lesions from rice rats of the different experimental groups. No significant differences were found between VEH/VEH and VEH/PTH rice rats (*p* = 0.446). However, ZOL/VEH rats had larger MRONJ-like lesions than VEH/VEH rats (*p* = 0.0031), with gingival ulceration and recession involving all three molars extending toward the palatal midline. Noteworthy, ZOL/PTH rats had smaller maxillary lesions than ZOL/VEH rats (*p* = 0.034).

### MicroCT analysis of maxillae

Figure 1C depicts representative 2D slices from MicroCT scans of the maxillary lesions of ZOL/VEH and ZOL/PTH rats. The quantitative Micro-CT analysis showed no differences in BV/TV, Tb.Th, BMD and TMD (Figures 1D,E,H,I) between ZOL/VEH and ZOL/PTH rats. In contrast, ZOL/PTH rice rats had lower Tb. N (*p* < 0.001; Figure 1F) and greater Tb.Sp (*p* < 0.001) than ZOL/VEH rats (Figure 1G).

### Histopathologic diagnosis of MRONJ and its prevalence

**Figure 2:**
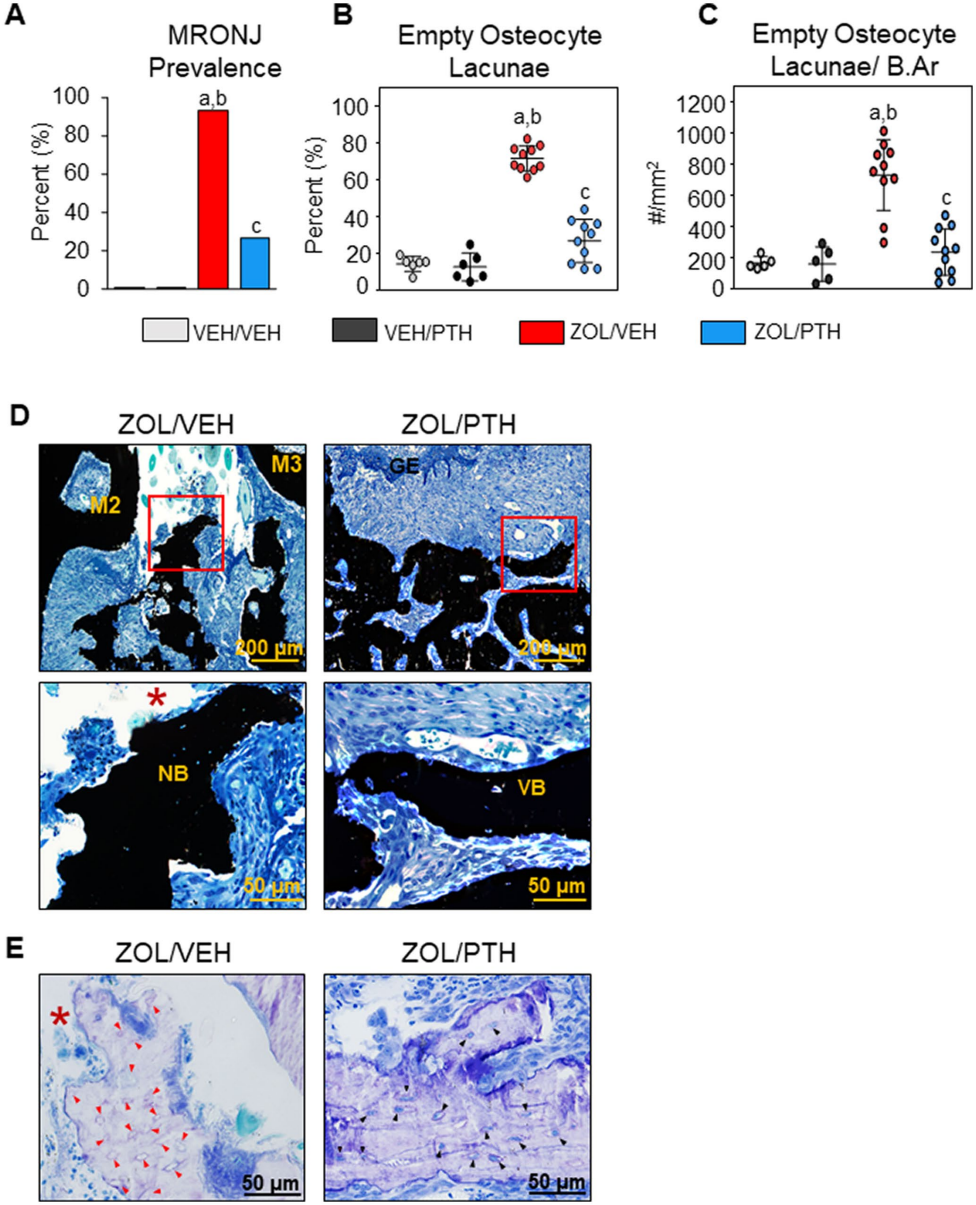
Histopathologic analysis of oral lesions. **(A)** Prevalence of MRONJ after 6 weeks treatment with saline or iPTH. **(B)** Percentage of empty osteocyte lacunae (%). **(C)** Number of empty osteocyte lacunae per bone area (#/mm^2^). **(D)** Representative photomicrographs of the interdental M2M3 lesion area in ZOL/VEH and ZOL/PTH groups stained with von Kossa and counterstained with tetrachrome. Exposed necrotic bone (red box, *) lacking overlying gingival epithelium and lamina propria can be seen in ZOL/VEH rats. In contrast, viable bone (red box) covered with gingival epithelium can be seen in ZOL/PTH rats. **(E)** Toluidine blue-stained sections of a ZOL/VEH rat shows exposed necrotic bone (*) with 10+ confluent empty osteocyte lacunae or osteocytes with pyknotic nuclei (red arrowheads). In contrast, ZOL/PTH rats show minimal empty osteocyte lacunae and large areas of vital bone with lacunae occupied by osteocytes with basophilic nuclei (black arrowheads).

Almost all (93%) of the ZOL/VEH rats with gross MRONJ-like lesions at the end of treatment were histopathologically confirmed to have MRONJ (Figure 2A). In contrast, only 27% of the ZOL/PTH rats with initial gross MRONJ-like lesions had histopathologic MRONJ at the end of treatment, with significantly less MRONJ prevalence than ZOL/VEH rats (Figure 2A). Furthermore, no MRONJ lesions were found in the VEH/VEH or the VEH/PTH groups.

In line with the MRONJ prevalence, we observed a lower percentage of empty osteocyte lacunae (*p* < 0.001) and the number of empty osteocyte lacunae/B.Ar (*p* < 0.001) in the involved maxillary bone of ZOL/PTH than ZOL/VEH rats (Figures 2B,C). In addition, very low values for these variables were found in VEH/VEH and VEH/PTH rats. Figure 2D depicts a panel of representative photomicrographs of sections stained with von Kossa counterstained with tetrachrome of the involved maxillary M2M3 area in ZOL/VEH and ZOL/PTH rice rats. The ZOL/VEH rat displays exposed necrotic bone, ulceration of the gingival epithelium and fibrosis of the lamina propria. In contrast, the ZOL/PTH rat shows vital alveolar bone with overlying gingival epithelium. Figure 2E depicts representative photomicrographs of sections stained with toluidine blue at the maxillary M2M3 area of ZOL/VEH and ZOL/PTH rats. Exposed alveolar bone with a greater number of empty osteocyte lacunae is present in the ZOL/VEH rat. In contrast, osteocyte lacunae are generally occupied by nuclei of viable osteocytes in the alveolar bone of the ZOL/PTH rat.

### Maxillary bone histomorphometry

**Figure 3:**
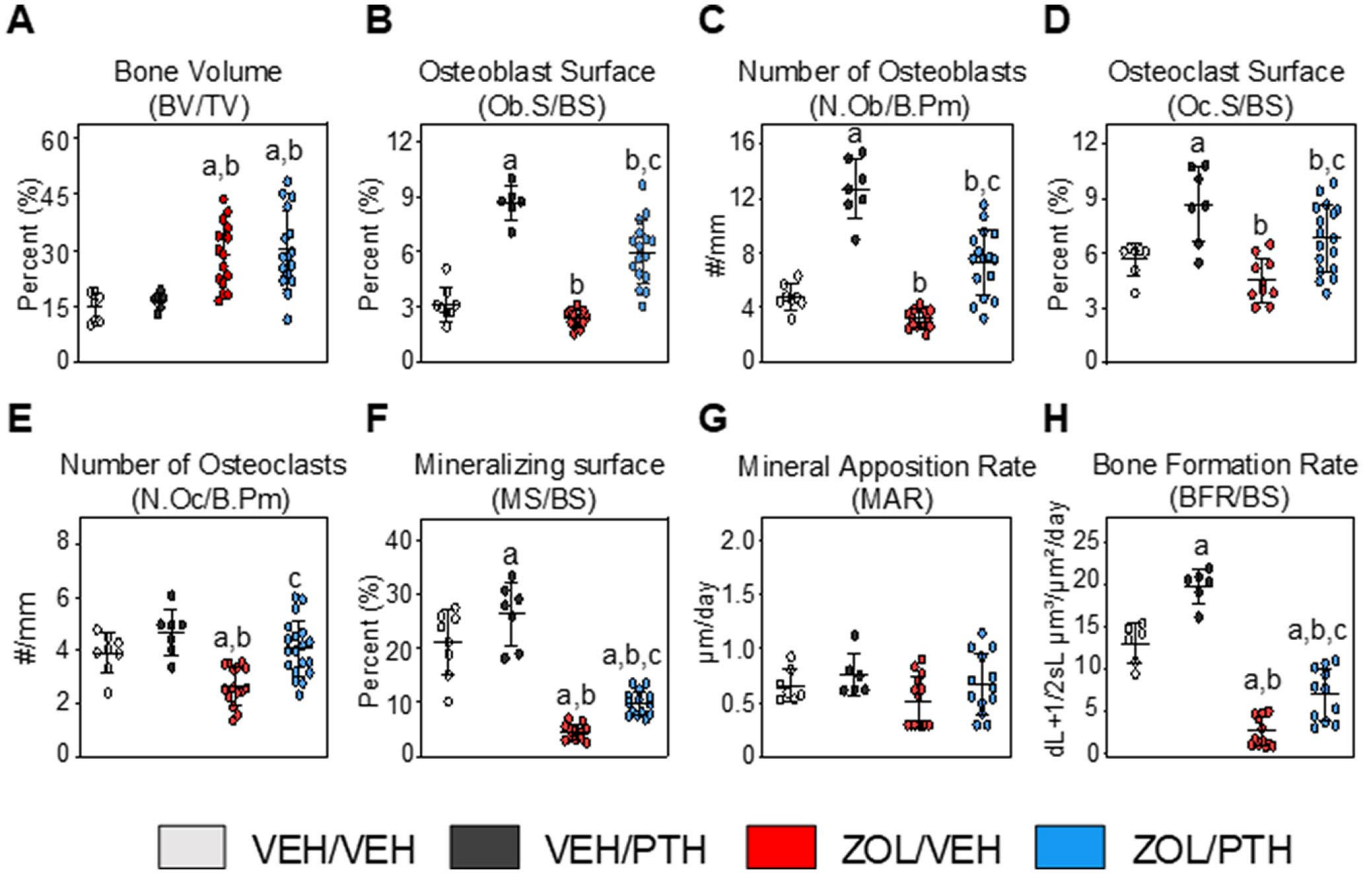
Assessment of bone volume and static and dynamic bone histomorphometric parameters at the maxillary M2M3 lesion area (ROI 1). **(A)** Bone volume (BV/TV). Static bone indices include. **(B)** Osteoblast surface per bone surface (Ob.S/BS). **(C)** Number of osteoblasts per bone perimeter (N.Ob/B.Pm). **(D)** Osteoclast surface per bone surface (Oc.S/BS). **(E)** Number of osteoclasts per bone perimeter (N.Oc/B.Pm). Dynamic bone indices include. **(F)** Mineralizing surface (MS/BS). **(G)** Mineral apposition rate (MAR). **(H)** Bone formation rate (BFR/BS). Data are expressed as Mean ± SD. Superscripts a–c denote significant differences from VEH/VEH, VEH/PTH and ZOL/VEH groups, respectively (*p* < 0.05).

*At ROI 1 (Lesion area):* BV/TV was greater in ZOL/VEH than VEH/VEH rats (*p* < 0.001) and in ZOL/PTH than VEH/PTH rats (*p* < 0.001; Figure 3A). However, no differences in BV/TV were found between ZOL/VEH and ZOL/PTH rats (*p* = 0.062). Interestingly, VEH/PTH rats had greater Ob.S/BS (*p* < 0.001) and N.Ob/B.Pm (*p* < 0.001) than VEH/VEH rats (Figures 3B,C). Likewise, these variables were greater in ZOL/PTH than in ZOL/VEH rats (*p* < 0.001, *p* < 0.001). Furthermore, no differences were observed in Oc.S/BS between ZOL/VEH and VEH/VEH rats (*p* = 0.085; Figure 3D). However, Oc.S/BS was greater in VEH/PTH than in VEH/VEH rats (*p* = 0.001). Similarly, greater Oc.S/BS was found in ZOL/PTH than in ZOL/VEH rats (*p* = 0.013; Figure 3D). The number of osteoclasts (N.Oc/B.Pm) was greater in ZOL/PTH than in ZOL/VEH rats (*p* < 0.001) and lower in ZOL/VEH than VEH/VEH (*p* < 0.001) and VEH/PTH (*p* < 0.001) rice rats, respectively (Figure 3E).

**Figure 4:**
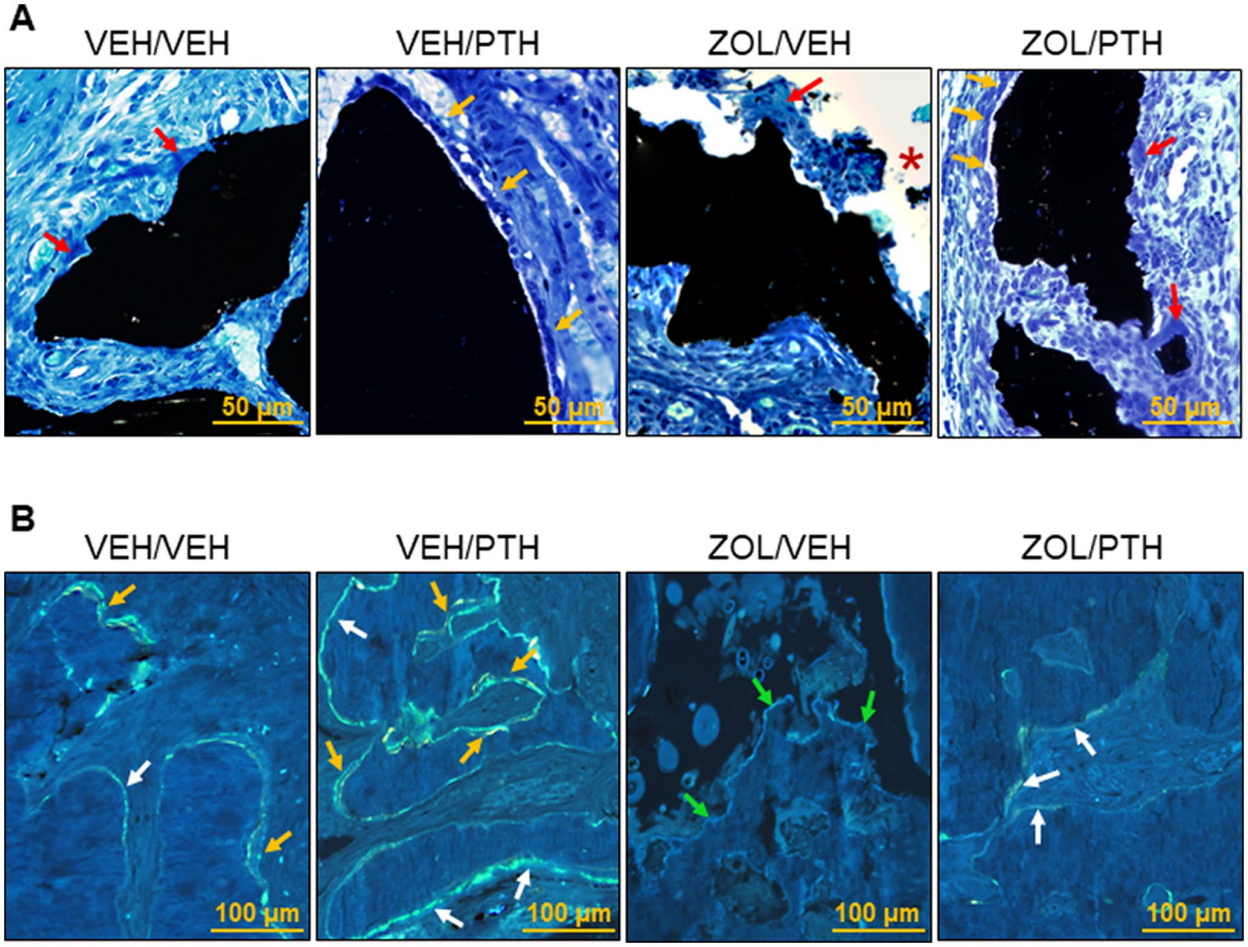
Representative photomicrographs of cancellous bone tissue at maxillary interdental M2M3 lesion area. **(A)** Four-um sections stained with von Kossa and counterstained with tetrachrome; magnification at 40×. Osteoclasts (red arrows) can be seen along the eroded surfaces in a VEH/VEH animal with localized periodontitis. Osteoblasts (yellow arrows) can be seen along the surface of the alveolar bone in a VEH/PTH rice rat. A ZOL/VEH rice rat shows exposed bone (*) lacking overlying gingival epithelium. Osteoblasts can be seen on the surface of cancellous bone (yellow arrows) in ZOL/PTH rice rats and regions of the eroded surface with osteoclasts (red arrows). **(B)** Fluorescent declomycin (yellow) and calcein (green) labels administered with a 5-day inter-label period are observed on the bone surface; magnification 200x. Exposed necrotic bone (green arrows) can be seen in the ZOL/VEH group without fluorochrome-labeled surfaces. Single-labels (white arrows) and double-labels (yellow arrows) can be seen along the surfaces of the alveolar bone in the VEH/VEH, VEH/PTH and ZOL/PTH groups.

In line with the static osteoblast findings, dynamic histomorphometry analysis showed greater MS/BS (*p* = 0.011) and BFR/BS (*p* < 0.001) in VEH/PTH than VEH/VEH rats and ZOL/PTH than ZOL/VEH rats (*p* = 0.001; *p* < 0.001), respectively (Figures 3F,H). However, no differences in MAR were found between these groups (*p* = 0.146; Figure 3G). In addition, MS/BS and BFR/BS were greater in VEH/PTH than in ZOL/PTH rats (*p* < 0.001, *p* = 0.001), respectively (Figures 3F,H). Figure 4A depicts representative photomicrographs of sections from ROI 1 of rats of the different groups stained with von Kossa counterstained with tetrachrome. Figure 4B depicts representative photomicrographs of unstained sections from ROI 1, showing single and double fluorochrome labels on alveolar bone surfaces.

*At ROI 2 (Non-affected area)*: VEH/PTH rats had greater BV/TV than VEH/VEH rats (*p* = 0.001; Supplemental Figure S3A). ZOL/VEH rats also had greater BV/TV than VEH/VEH rats (*p* < 0.001). However, no differences in BV/TV were found between ZOL/VEH and ZOL/PTH rats. Furthermore, VEH/PTH rats had greater Ob.S/BS (*p* < 0.001) and N.Ob/B.Pm (*p* < 0.001) than VEH/VEH rats. Likewise, ZOL/PTH rats had greater Ob.S/BS (*p* < 0.001) and N.Ob/B.Pm (*p* < 0.028) than ZOL/VEH rats, but both endpoints were lower than VEH/PTH rats (*p* = 0.003, *p* = 0.023; Supplemental Figures S3B,C).

Oc.S/BS (*p* = 0.004) and N.Oc/B.Pm (*p* = 0.019) were greater in ZOL/PTH than in ZOL/VEH rats.

Furthermore, Oc.S/BS (*p* = 0.004) and N.Oc/B.Pm (*p* = 0.019) were lower in ZOL/VEH than in ZOL/VEH rats (Supplemental Figures S3D,E). In addition, N.Oc/B.Pm was greater in VEH/PTH than VEH/VEH rats (*p* = 0.027), but no differences in N.Oc/B.Pm was found between both groups (*p* = 0.438; Supplemental Figures S3D,E).

Dynamic histomorphometry showed that ZOL/VEH rats have lower MS/BS (*p* < 0.001), MAR (*p* < 0.001) and BFR/BS (*p* < 0.001) than VEH/VEH rats (Supplemental Figures S3F–H). Similarly, lower MS/BS (*p* < 0.001) and BFR/BS (*p* = 0.032) were seen in ZOL/PTH than in VEH/PTH rats. Furthermore, greater MS/BS (*p* = 0.004), MAR (*p* = 0.006) and BFR/BS (*p* < 0.032) were found in ZOL/PTH than in ZOL/VEH rats. In addition, greater MS/BS was observed in VEH/PTH than in VEH/VEH rats (*p* = 0.012); however, no differences in MAR and BFR/BS were seen between these groups (Supplemental Figures S3F–H).

### Oral/gingival epithelium thickness and proliferation

#### Oral/gingival epithelial thickness

**Figure 5:**
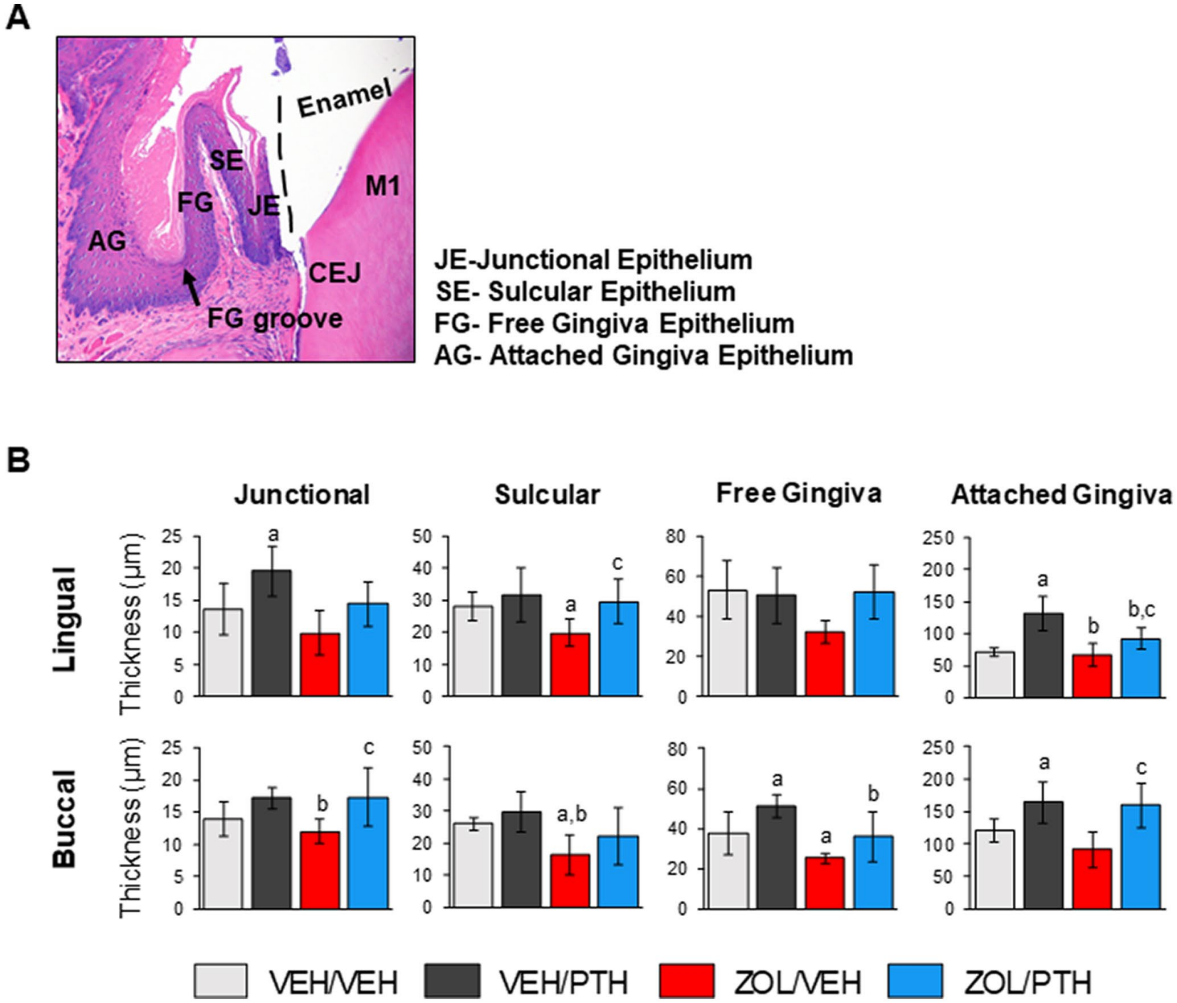
Effects of ZOL and iPTH on the gingival epithelial thickness of the mandible. **(A)** Representative photomicrograph of an H&E-stained section of the gingival epithelium in the mandible at the lingual side of the M1 region. **(B)** Gingival epithelial thickness was evaluated at four different regions, including the junctional, sulcular, free gingiva and attached gingival epithelium at the lingual and buccal surfaces. Bars represent mean ± SD. Superscripts a–c denote significant differences from VEH/VEH, VEH/PTH and ZOL/VEH rice rats, respectively (*p* < 0.05).

The effects of ZOL and iPTH treatment on healthy gingival epithelium were assessed in the mandible at the four different regions depicted in Figure 5A. We found lower epithelial thickness of the sulcular gingiva at the lingual (*p* = 0.033) and buccal (*p* = 0.021) surfaces in ZOL/VEH compared to VEH/VEH rats (Figure 5B). Furthermore, ZOL/VEH rice rats had thinner free gingiva epithelium at the buccal surface than VEH/VEH rats (Figure 5B). We also found that VEH/PTH rats have thicker gingival epithelium at the lingual surface of the junctional (*p* = 0.029) and attached gingiva (*p* < 0.001; Figure 5B) and at the buccal surface of the free gingiva (*p* = 0.015) and attached gingiva (*p* = 0.023) compared to VEH/VEH rats (Figure 5B). Moreover, we observed thicker epithelium at the lingual surface of the sulcular (*p* = 0.018) and attached gingiva (*p* = 0.038) in ZOL/PTH than in ZOL/VEH rats (Figure 5B). In addition, greater epithelial thickness was found at the buccal surface of the junctional (*p* = 0.007) and attached gingiva (*p* < 0.001) in ZOL/PTH rats than in ZOL/VEH rats (Figure 5B).

#### Oral/gingival epithelial proliferation

**Figure 6:**
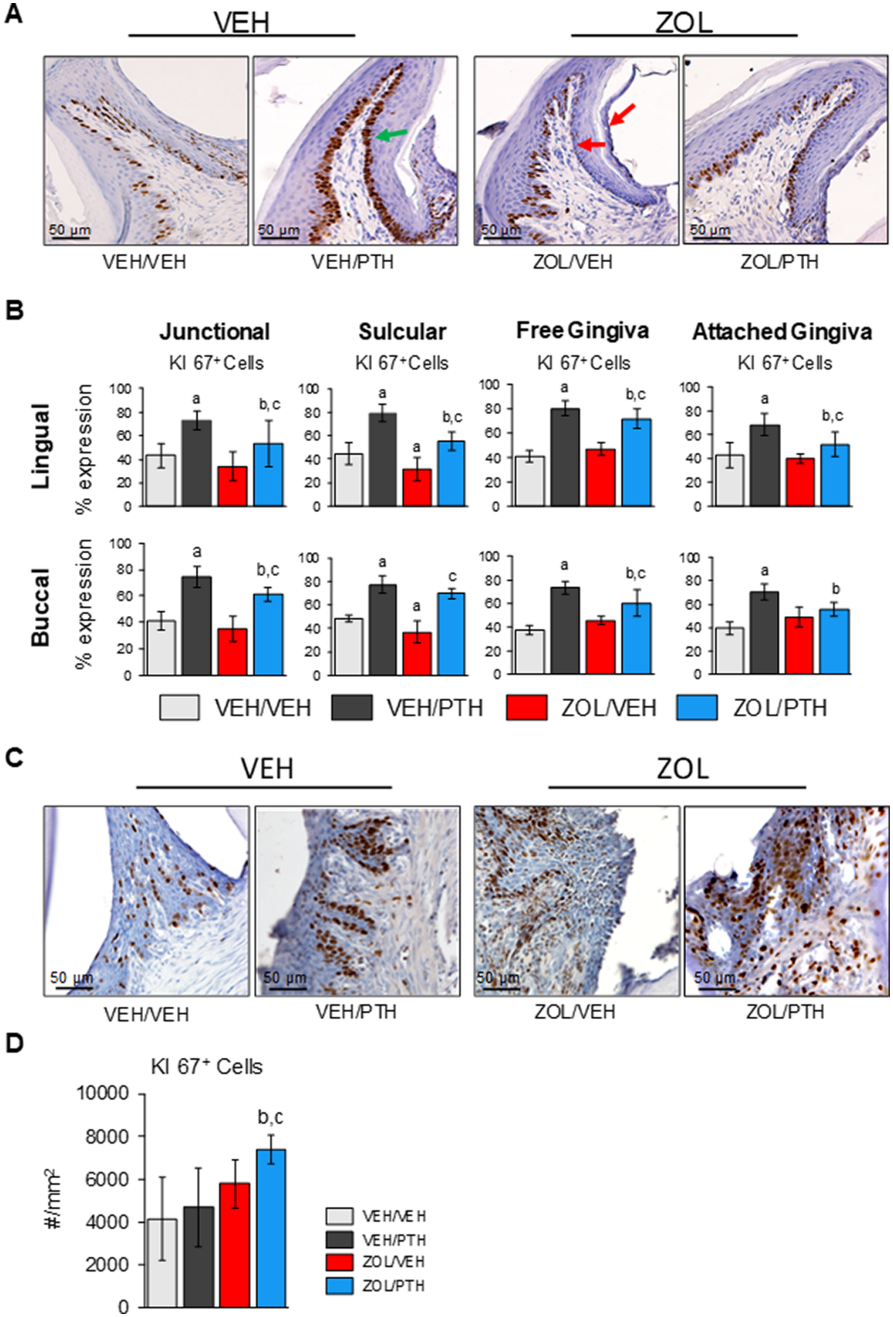
Effects of ZOL and iPTH on regenerative capacity on the gingival epithelium of the mandible (non-lesion area) and maxilla (lesion area). **(A)** Representative photomicrographs of brown stained Ki67^+^ cells in the gingival epithelium of the M1 region of the mandible. The green arrows show high proliferation. Red arrows indicate the low proliferation of the basal cells. **(B)** The percentage of proliferating cells was evaluated at four different regions, including junctional, sulcular, free gingiva, and attached oral epithelium on the lingual and buccal surfaces. Superscripts a–c denote significant differences from VEH/VEH, VEH/PTH, and ZOL/VEH, respectively. **(C)** Representative photomicrographs of brown stained Ki 67^+^ cells in the gingival epithelium surrounding the lesion area. **(D)** The number of proliferating cells per tissue area was evaluated at the lingual aspect of the maxillary M2M3 interdental lesion’s gingiva.

Figure 6A depicts representative photomicrographs of Ki67^+^ epithelial cell expression at the basal two layers of the four gingival regions in the mandible. We found fewer Ki67^+^ epithelial cells at the sulcular epithelium, both lingual (*p* = 0.012) and buccal (*p* = 0.008) surfaces, in ZOL/VEH than in VEH/VEH rats (Figure 6B). In contrast, more Ki67^+^ epithelial cells were observed at the lingual and buccal surfaces of the junctional (*p* = 0.002, *p* < 0.001), sulcular (*p* < 0.001; *p* < 0.001), free gingiva (*p* < 0.001; *p* = 0.002) and attached gingiva (*p* < 0.001; *p* < 0.001) of VEH/PTH than VEH/VEH rats (Figure 6B). Furthermore, more Ki67^+^ epithelial cells were found at lingual and buccal surfaces of the junctional (*p* = 0.025, *p* < 0.001), sulcular (*p* < 0.001, *p* < 0.001) and free gingiva epithelia (*p* < 0.001, *p* = 0.002) in ZOL/PTH than in ZOL/VEH rats. In addition, more Ki67^+^ epithelial cells were found at the lingual surface of the attached gingival epithelium in ZOL/PTH than in ZOL/VEH rats (*p* = 0.026; Figure 6B).

Figure 6C depicts representative photomicrographs of Ki67^+^ epithelial cell expression in the gingiva of the maxillary ROI 1. We found no differences in the number of Ki67^+^ epithelial cells between VEH/VEH and VEH/PTH rats and ZOL/VEH and VEH/VEH rats, respectively (Figure 6D). Furthermore, we found more Ki67^+^ epithelial cells in ZOL/PTH than in ZOL/VEH rats (*p* = 0.036). In addition, more Ki67^+^ epithelial cells were found in the ZOL/PTH than in VEH/PTH rats (*p* < 0.001).

### Quantification of T-cells and neutrophils in oral soft tissues

**Figure 7:**
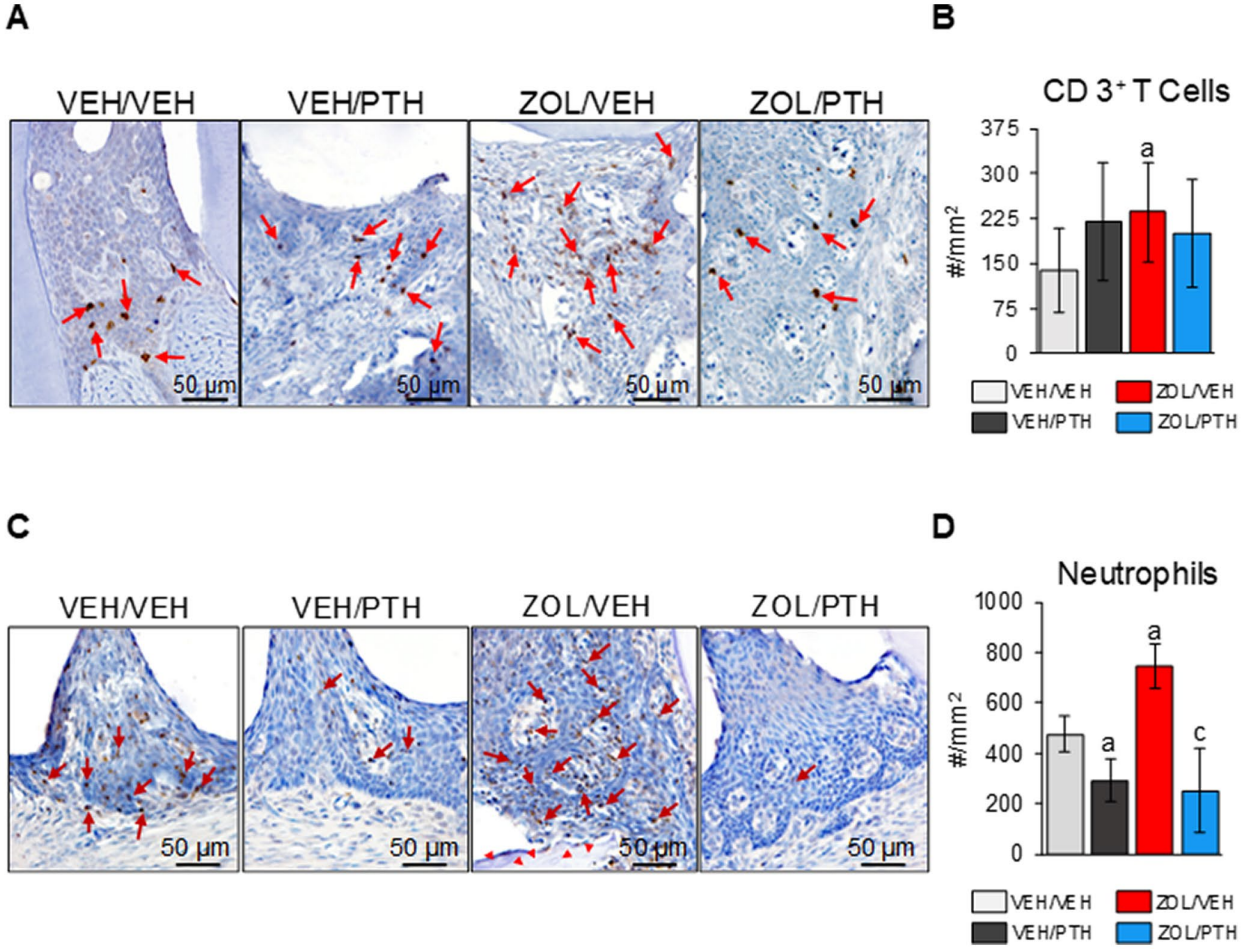
Assessment of CD 3^+^ T cells and neutrophils at the M2M3 interdental lesion area. **(A)** Representative photomicrographs showing CD 3^+^ T cells (red arrows) in the gingival epithelium, lamina propria, and underlying connective tissue. **(B)** Number of CD 3^+^ T cells per lesion area (#/mm^2^). **(C)** Representative photomicrographs showing neutrophils (red arrows) in the gingival epithelium and lamina propria. Red arrowheads show necrotic bone in the ZOL/VEH group. **(D)** Number of neutrophils per lesion area (#/mm^2^). Superscripts a and c denote significant differences from VEH/VEH and ZOL/VEH rice rats, respectively (*p* < 0.05).

Figure 7A depicts representative photomicrographs of the expression of CD3^+^ T cells at the maxillary ROI 1. We found more CD3^+^ T cells in the MRONJ lesions of ZOL/VEH than in the periodontal lesions of VEH/VEH rats (Figure 7B). Furthermore, we found no differences in the number of CD3^+^ T-cells between oral lesions in ZOL/PTH and ZOL/VEH rats and between VEH/VEH and VEH/PTH rats, respectively (Figure 7B). Figure 7C depicts representative photomicrographs of the presence of neutrophils at the maxillary ROI 1. ZOL/VEH rats with MRONJ had more neutrophils than VEH/VEH rats with periodontitis (*p* = 0.001; Figure 7D). In contrast, VEH/PTH rats had fewer neutrophils at the lesion site than VEH/VEH rats (*p* = 0.017). Similarly, ZOL/PTH rats had fewer neutrophils than ZOL/VEH rats (*p* < 0.001; Figure 7D).

## Discussion

This experiment demonstrates a new use for an established, infection-based pre-clinical model of MRONJ (18, 20, 21, 74, 78). We treated rice rats that were in the process of developing localized periodontal lesions with a vehicle or an oncology dose of ZOL for 30 ± 10 weeks. In past studies, rice rats exposed to this combination of systemic (ZOL) and local (developing localized periodontitis) factors for 18–24 weeks exhibited a 70–100% prevalence of MRONJ (18, 21, 74, 78). Using bi-weekly *in vivo* oral examination, we identified and subsequently tracked evolving localized oral lesions in the ZOL- and vehicle-treated rats. When an oral lesion similar to those in past studies had persisted for at least 8 weeks with sufficient severity to be termed an MRONJ-like lesion, we continued vehicle or ZOL and began 6 weeks of treatment with iPTH. On treatment, MRONJ-like lesions in ZOL-treated rice rats not given iPTH enlarged, while those in ZOL-treated rats given iPTH tended to shrink or did not enlarge. At the end of the study, ZOL-treated rats with iPTH had smaller oral lesions than ZOL-treated rats without iPTH. Furthermore, at the end of the study, while 93% of ZOL-treated rats receiving vehicle had histologically-verified MRONJ, only 27% of ZOL-treated rats receiving iPTH had histologically-verified MRONJ (*p* < 0.01).

Our non-invasive procedure to identify an MRONJ-like lesion that is ready for treatment here basically resembles the algorithm currently used to diagnose MRONJ in humans. As in humans, when an oral lesion with a similar location and gross characteristics to those generally associated with necrotic alveolar bone in the underlying tissue exists for 8 weeks, a diagnosis of MRONJ is made. As in humans newly diagnosed with MRONJ, no realistic possibility exists here to histologically verify necrotic bone in each lesion in ZOL-treated animals at the start of treatment. We believe that, at this point, it is reasonable to assume from past data of this model that necrotic alveolar bone existed in the localized lesions of each ZOL-treated rice rat at the start of the iPTH treatment. Future experiments of this type might include a non-invasive imaging modality such as *in vivo* MicroCT or μMRI to clarify the presence of necrotic alveolar bone further when treatment begins and perhaps also track its evolution.

Our study demonstrates that 6 weeks of iPTH treatment enhances the healing of established MRONJ-like lesions in rice rats. While 93% of ZOL/VEH rats had MRONJ, only 27% of the ZOL/PTH rats had MRONJ after treatment, representing a 70% decrease in MRONJ. Consistent with this decrease, there were significantly fewer empty osteocyte lacunae in the involved maxillary alveolar bone area in ZOL/PTH rats than in ZOL/VEH rats. Furthermore, iPTH-treated ZOL rats had smaller oral lesions than vehicle-treated ZOL rats. While MRONJ-like lesions in ZOL/VEH rats continued to enlarge during the treatment period, MRONJ-like lesions in ZOL/PTH rats stabilized. ZOL/PTH rats also exhibited a higher bone turnover rate, implying a higher necrotic alveolar bone removal rate than in ZOL/VEH rats. ZOL/PTH rats also showed a higher gingival epithelial proliferation rate and lower inflammation in the oral lesion tissue.

Most preclinical studies have examined iPTH as a preventative therapy for tooth extraction-related MRONJ. When given before the development of MRONJ and immediately after a tooth extraction, iPTH treatment is promising. It enhances bone healing of the socket, promotes soft tissue coverage, and reduces the incidence of MRONJ (43, 44, 46, 47). However, only a few preclinical studies investigated the effects of iPTH in established MRONJ lesions (50, 51, 93, 94). Indeed, Zandi et al. (50) showed that PTH dose-dependently improved clinical and histologic oral lesion variables in Wistar rats that developed MRONJ after tooth extraction. Furthermore, Ersan et al. (93) found that iPTH reduces the area of bone necrosis of MRONJ lesions in ZOL-treated Sprague Dawley rats following tooth extraction. Liu et al. (51) also showed that iPTH reduced the area of necrotic bone and the number of empty osteocyte lacunae in MRONJ lesions following tooth extraction in ovariectomized Sprague Dawley rats treated with ZOL and dexamethasone (DEX). In addition, Yu and Su (94) showed that PTH (3, 10 or 30 μg/kg) reduced the incidence of MRONJ in C57BL/6 mice treated with ZOL/DEX for 16 weeks after tooth extraction. Similar to these studies, our experiment indicates that iPTH is a promising treatment for established MRONJ, this time using an MRONJ model that involves infection.

This study provides unique data that further supports and elucidates the action of iPTH on bone healing surrounding MRONJ. The comprehensive histomorphometric analysis shows that the turnover rate in alveolar bone is higher in ZOL-treated rats given iPTH than in ZOL-treated rats not given iPTH. We found that iPTH increases multiple histomorphometric bone resorption and formation variables at the lesion site. First, we found that iPTH affected osteoclasts and stimulated bone resorption in the vehicle- and ZOL-treated rats. Osteoclast surface was greater in VEH/PTH than in VEH/VEH rats and ZOL/PTH than in ZOL/VEH rats. Furthermore, osteoclast number was greater in ZOL/PTH than in ZOL/VEH rats. Second, we found that iPTH stimulated bone formation. Greater osteoblast surface, osteoblast number, mineralizing surface and bone formation rate were seen in VEH/PTH than in VEH/VEH rice rats and ZOL/PTH rats than in ZOL/VEH rice rats. The bone anabolic effect of iPTH was partially blunted by ZOL, as indicated by the significantly lower values for these histomorphometric variables in ZOL/PTH compared to VEH/PTH rats. Though Micro CT showed no differences in bone volume at the lesion area of ZOL/VEH and ZOL/PTH rats, the trabecular number was lower in ZOL/PTH than in ZOL/VEH rats. The lower trabecular number may be due to the removal of trabeculae containing necrotic bone in ZOL/PTH rats that are not removed in ZOL/VEH rats.

Altogether, our data suggest that iPTH promotes the remodeling of alveolar bone, positively affecting bone healing in MRONJ. We believe it can be readily inferred that in ZOL-treated rats that had developed necrotic alveolar bone, inducing a high turnover state in alveolar bone caused the removal of a significant amount of that necrotic bone and its replacement with new viable bone within 6 weeks. Furthermore, our study provides insight into the healing effect of iPTH on soft tissue. MRONJ lesions with the exposed bone are almost always accompanied by ulceration of the oral epithelia and underlying soft tissues. It has been proposed that when N-BPs are released to the oral microenvironment from the alveolar bone matrix surrounding the MRONJ lesion as a result of an event involving inflammatory bone resorption (e.g., inflammatory dental disease, oral trauma, invasive surgeries, etc.), induce toxic effects to the overlying soft tissues, including the oral epithelia, and alter oral soft tissue homeostasis and its healing capacity (57, 95). In addition, N-BPs reduce proliferation, number and migration and increase cell cycle arrest of oral keratinocytes (96–99). N-BPs have also been demonstrated to reduce epithelial thickness in a 3D oral mucosa model containing oral fibroblasts and keratinocytes on a scaffold analogous to *in vivo* tissue (100).

In this study, the ZOL-treated rat groups had lesser epithelial thickness and proliferative capacity of their healthy gingival epithelium, particularly at the sulcular region, than their respective VEH control rat groups. However, iPTH-treated rats had greater epithelial thickness and proliferative capacity in the absence and presence of ZOL administration in most of the gingiva/oral epithelium regions. In addition, the response of the oral epithelium to PTH was significantly less in the presence of ZOL than in the absence of ZOL. Contrasting findings have been reported in an *in vitro* study, in which treatment with PTH (1–34) inhibited the proliferation of keratinocytes in a dose-dependent manner (65). The differences in the proliferative capacity may be due to the mode in that PTH is administered, considering that continuous and intermittent administration of PTH has contrasting catabolic and anabolic effects on bone (101–103). In a separate study, intermittent versus continuous PTH (1–34) was assessed in human periodontal ligament cell proliferation and survival *in vitro* (104). Low-dose exposure to iPTH enhanced proliferation in periodontal ligament cell cultures, something that continuous PTH did not do.

iPTH reduced inflammation in the localized periodontitis or MRONJ lesion. Indeed, VEH/PTH rice rats had fewer neutrophils than VEH/VEH rice rats with periodontitis, and ZOL/PTH rice rats had fewer neutrophils than ZOL/VEH rats with MRONJ. Thus, iPTH may play an anti-inflammatory role by reducing the number of neutrophils at the MRONJ lesion site preventing further tissue destruction through the regeneration of reactive oxygen species and pro-inflammatory cytokines (105).

Together, these findings support further experimentation with PTH for treating MRONJ in non-cancer patients. They may also suggest that any agent that elevates bone turnover, even transiently, can be useful in treating MRONJ by driving the removal of necrotic alveolar bone. These data suggest that iPTH treatment would also be effective in denosumab-induced MRONJ. Currently, most literature consists of case studies in osteoporosis patients treated with teriparatide to resolve MRONJ with positive results (31, 32, 34, 106). However, it is important to remember that PTH is contraindicated in patients with cancer because it increases bone remodeling, possibly contributing to bone metastasis (107). In addition, clinical studies in patients with severe osteoporosis showed that Teriparatide in combination with denosumab induced higher BMD gains and was more effective than each agent alone, as occurs with the combination of Teriparatide and N-BPs (108–111).

We recognize the limitations of this study. One of these is that we did not use invasive or non-invasive *in vivo* approaches, such as MicroCT or μMRI, to identify oral necrotic bone at baseline and throughout treatment in the rice rats with MRONJ lesions. Another limitation is that for evaluating inflammation in the MRONJ and periodontitis lesions, we limited the investigation to quantifying CD3^+^ and neutrophils in the affected oral tissues and performing tissue histopathology. A more comprehensive evaluation, including other different inflammatory cell types and the subpopulations of CD3^+^ cells, would be advantageous to understand oral inflammation in MRONJ.

## Conclusion

Our data suggest that iPTH is an efficacious non-operative therapy that enhances the resolution of the hard and soft tissue pathology in established MRONJ lesions in a validated preclinical animal model of MRONJ.

## Data availability statement

The raw data supporting the conclusions of this article will be made available by the authors, without undue reservation.

## Ethics statement

The animal study was reviewed and approved by IACUC.

## Author contributions

EC performed most of the experiments under the supervision of JA. EC analyzed the results and wrote the manuscript with JA. JJ, CCr, DF, and CCa contributed to the attainment of data and preparation of samples for analysis. JY helped with *ex vivo* micro CT scanning and analysis. IB and DK gave feedback on the manuscript and experimental procedure. JA designed the experimental procedure, performed experiments himself, supervised EC, co-analyzed the results with EC and wrote the manuscript. All authors contributed to the article and approved the submitted version.

## Funding

This research was supported by the National Institute of Dental and Craniofacial Research (NIDCR); R01DE023783-01A.

## Conflict of interest

The authors declare that the research was conducted in the absence of any commercial or financial relationships that could be construed as a potential conflict of interest.

## Publisher’s note

All claims expressed in this article are solely those of the authors and do not necessarily represent those of their affiliated organizations, or those of the publisher, the editors and the reviewers. Any product that may be evaluated in this article, or claim that may be made by its manufacturer, is not guaranteed or endorsed by the publisher.

## Abbreviations

MRONJ: medication-related osteonecrosis of the jaw
pARs: powerful antiresorptives
N-BPs: nitrogen-containing bisphosphonates
ZOL: zoledronic acid
PTH: parathyroid hormone
iPTH: intermittent PTH
STD: standard diet
DEX: dexamethasone
GQG: gross quadrant grades
BW: body weight
M: molar
VEH: vehicle
Le. Ar: lesion area
Tt.Mx.Ar: total maxillary area
ROI: region of interest
BV/TV: bone volume/total volume
Tb. Th: trabecular thickness
Tb. N: trabecular number
Tb. S: trabecular separation
N. Ob/B.Pm: osteoblast number/alveolar bone perimeter
Ob.S/BS: osteoblast surface
N.Oc/B.Pm: osteoclast number/alveolar bone perimeter
Oc.S/BS: osteoclast surface
sL/BS: single-labeled surfaces
dL/BS: double-labeled surfaces
MS/BS: mineralizing surface
MAR: mineral apposition rate
BFR/BS: bone formation rate
