# Supplementary material for: Intermittent parathyroid hormone enhances the healing of medication-related osteonecrosis of the jaw lesions in rice rats
Source: Front Med (Lausanne). 2023 Jun 19;10:1179350. doi: 10.3389/fmed.2023.1179350 (PMC10315582; doi:10.3389/fmed.2023.1179350)
Supplement: Supplementary file 1 [file Table_1.DOCX]

Supplementary Material

**Intermittent parathyroid hormone enhances the healing of medication-related osteonecrosis of the jaw lesions in rice rats**

E.J. Castillo^1^, J.M. Jiron^1^, C S. Croft^1^, D.G. Freehill^1^, C.M. Castillo^1^, J, Kura^2^, J.F. Yarrow^2^, I. Bhattacharyya^3^, D.B. Kimmel^1^, J.I. Aguirre^1*^

^1^ Department of Physiological Sciences, University of Florida (UF), Gainesville, FL;

^2^ VA Medical Center, Research Service, VA Medical Center, Gainesville, FL.

^3^ Department of Oral & Maxillofacial Diagnostic Sciences, College of Dentistry, UF

* Please address correspondence to:

J. Ignacio Aguirre, D.V.M., Ph.D., DACLAM

Department of Physiological Sciences

Box 100144, JHMHC

University of Florida

Gainesville, FL 32610

Telephone: (352) 294-4038

FAX: (352) 392-5145

E-mail: [aguirrej@ufl.edu](mailto:aguirrej@ufl.edu)

**Supplemental Table 1. Criteria for Gross Quadrant Grades (*GQG*)**

| **GQG** | **Degree** | **Gross Lesion Description** |
| --- | --- | --- |
| **0** | Absence | None |
| **0.5** | Incipient | Hair strands and food particles in an interdental space with no soft tissue inflammation. |
| **1** | Slight | A discrete lesion in an interdental space; minimal gingival recession; impacted materials (hair, food debris); inflammation at the margin of impacted materials. |
| **2** | Mild | Single open wound lesion in an interdental space with ulceration/ recession of lingual gingiva along 1/2 to 2/3 the lingual gingival margin of one involved molar; limited extension into the lingual mucosa; inflammation/swelling/redness at the margin of impacted material; or presence of two separate *GQG*=1 lesions (one always lingual, the other lingual or buccal) in the same quadrant. |
| **3** | Moderate | Open wound involving gingival recession/ulceration along the entire lingual gingival margin of two molars; ulceration extending into lingual mucosa towards the midline in the maxilla or onto the lingual plate (mandible); inflammation/ swelling/redness at the margin of impacted material; possible involvement of buccal mucosa. |
| **4** | Severe | Open wound involving gingival recession/ulceration around all three molars; ulceration extending into lingual mucosa toward the midline in the maxilla or onto the lingual plate (mandible) and the buccal mucosa; marked gingival inflammation/swelling/ redness along the lingual gingival margin of all molars; possible tooth migration or loss. |

**Supplemental Figure 1.**

**Experimental Design.** Eighty-four rice rats of both genders, age 4 wks, were fed a standard (STD) rodent chow diet to induce localized periodontitis. Animals were then randomized to receive either 0.9% VEH saline or zoledronic acid, ZOL (80 µg/kg BW) intravenously (IV) q 4 wks until the end of the study. Oral exams were performed biweekly in all rats to determine the occurrence and progression of lesions. The oral lesion induction period was ~30±10wks. VEH-treated rice rats only developed maxillary localized periodontitis. Rats that developed periodontitis and were treated with ZOL had lesions that evolved into MRONJ-like lesions at the end of the induction period. VEH and ZOL-treated rats with PD or MRONJ-like lesions were further randomized into subgroups. The VEH-treated rice rats were further randomized into two subsets: one subset received 0.9% saline (***VEH/VEH***), and the other subset received human PTH (1-34; teriparatide; 40μg/Kg [Bachem, Torrance, CA, USA] subcutaneously (SC) 3 times/wk for 6 wks (***VEH/PTH***). The ZOL-treated rice rats with MRONJ-like lesions were also further randomized into two subsets: One subset received 0.9% saline SC 3 times/wk for 6 wks (***ZOL/VEH)***. The other subset received PTH SC 3 times/wk for 6 wks (***ZOL/PTH)***. After 6 wks of treatment with saline or PTH, rats were euthanized, and jaws were collected for gross, histologic, static and dynamic histomorphometry analyses.

**Supplemental Data for Supplemental Figure 2**

***PQCT Analysis of Femoral Metaphysis and Mid-diaphysis***

Total metaphyseal BMC (*p<0.001,* *p<0.001*) and BMD (*p<0.001*, *p<0.001*) were greater in ZOL/VEH than in VEH/VEH male and female rice rats, respectively (**Supplemental Figure 2A**). Similarly, total metaphyseal BMC (*p=0.046*, *p=0.003*) and BMD *(p=0.003, p=0.006*) were greater in ZOL/PTH group than in ZOL/VEH male and female rice rats. Furthermore, total metaphyseal BMD was greater in VEH/PTH than in VEH/VEH male and female rice rats, respectively (*p=0.002*, *p<0.001*) (**Supplemental Figure 2B**). In line with these findings, mid-diaphyseal BMC and BMD were greater in ZOL/VEH than in VEH/VEH male and female rice rats (*p<0.001, p<0.001), respectively* (**Supplemental Figure 2C**). Further, mid-diaphyseal BMC was greater in VEH/PTH and then VEH/VEH male and female rice rats (*p=0.010*, *p=0.012*), respectively. In addition, mid-diaphyseal BMC was greater in ZOL/PTH than VEH/PTH male rice rats (*p=0.028*), and mid-diaphyseal BMD was greater in VEH/PTH than VEH/VEH female rice rats (*p<0.001*) (**Supplemental Figure** **2D**). However, no difference was observed in mid-diaphyseal BMD between ZOL/VEH and ZOL/PTH rice rats.

**Supplemental Figure 2.** Assessment of pQCT bone parameters of the femoral metaphysis and mid-diaphysis. **A**. Total metaphysis BMC. **B**. Total Metaphysis BMD. **C**. Mid-diaphyseal BMC. **D**. Mid-diaphyseal BMD. Bars indicate mean ± SD. Black superscripts a, b and c denote significant differences from VEH/VEH, VEH/PTH and ZOL/VEH male rice rats, respectively (p<0.05). Red superscripts a, b and c denote significant differences from VEH/VEH, VEH/PTH and ZOL/VEH female rice rats, respectively (p<0.05).

**Supplemental Figure 3.** Assessment of bone volume and static and dynamic bone histomorphometry parameters at the distal M3 non-lesion area (ROI 2). **A**. Percentage of bone volume (BV/TV). Static indices include **B**. Percentage of osteoblast surface per bone surface (Ob.S/BS), **C**. Number of osteoblasts per bone perimeter (N.Ob/B.Pm), **D**. Percentage of osteoclast surface per bone surface (Oc.S/BS) and **E**. Number of osteoclasts per bone perimeter (N.Oc/B.Pm). Dynamic indices include **F**. Mineralizing surface (MS/BS), **G**. Bone formation rate (BFR/BS) and **H**. Mineral apposition rate (MAR). Bars indicate mean ± SD. Superscripts a, b and c denote significant differences from VEH/VEH, VEH/PTH and ZOL/VEH rice rats, respectively (p<0.05).
